# Clinical perspectives on vagus nerve stimulation: present and future

**DOI:** 10.1042/CS20210507

**Published:** 2022-05-10

**Authors:** Eibhlin Goggins, Shuhei Mitani, Shinji Tanaka

**Affiliations:** 1 Division of Nephrology and Center for Immunity, Inflammation, and Regenerative Medicine, University of Virginia, Charlottesville, VA, U.S.A.; 2 Division of Nephrology and Endocrinology, The University of Tokyo Graduate School of Medicine, Tokyo, Japan

**Keywords:** neuro–immune interactions, optogenetics, vagus nerve stimulation

## Abstract

The vagus nerve, the great wanderer, is involved in numerous processes throughout the body and vagus nerve stimulation (VNS) has the potential to modulate many of these functions. This wide-reaching capability has generated much interest across a range of disciplines resulting in several clinical trials and studies into the mechanistic basis of VNS. This review discusses current preclinical and clinical evidence supporting the efficacy of VNS in different diseases and highlights recent advancements. Studies that provide insights into the mechanism of VNS are considered.

## Introduction

The word vagus is Latin for ‘wandering’, a name this exceptional nerve fully deserves. The vagus nerve (VN), the tenth cranial nerve, is the longest of the cranial nerves and has the most complex and diverse functions. One can envision the vagus wandering throughout the body, affecting numerous processes in its tracts. The VN is involved in regulation of the autonomic, immune, cardiovascular, gastrointestinal, respiratory and endocrine systems.

The VN is a mixed nerve composed of 20% efferent fibers and 80% afferent fibers and it serves as a bidirectional communicator between the brain and body [1]. Efferent functions include sending parasympathetic cholinergic signals, originating from the nucleus ambiguus and dorsal motor nucleus (DMN), to target organs including the lungs, digestive tract, and heart [2–5]. There are three afferent VN types including general somatic afferent (GSA), general visceral afferent (GVA), and special visceral afferent (SVA). These afferents transmit ascending sensory information and terminate in four vagal nuclei located within the medulla including nucleus of the solitary tract (NTS), the nucleus ambiguus, the trigeminal spinal nucleus and DMN [6].

The use of vagus nerve stimulation (VNS) can be first credited to American neurologist James Corning who attempted the technique in the 1880s for treatment of epilepsy [7]. His idea, which was based on evidence suggesting increased blood flow to the brain caused seizures, was largely abandoned for many years due to inconsistent results but resurfaced again in the 1900s [7]. While Corning focused on the indirect physiological effects of VNS, Bailey and Bremer, in the 1930s, investigated the direct effects of VNS on the central nervous system (CNS) [8]. These investigations led to the observation that VNS causes electroencephalogram (EEG) changes. Throughout the rest of the century, various animal studies utilizing VNS were conducted, but it was not until the 1990s that these transitioned into clinical studies. In 1988, the first implanted VNS device in a human was reported [9]. In 1997, the Food and Drug Administration (FDA) approved the first implantable VNS device for treating refractory epilepsy. Since this time, the FDA has approved the use of VNS for depression, migraines and cluster headaches, and in the abdomen for obesity. In this review, we focus on the current uses of VNS, potential applications and recent advancements in the field of VNS including auricular VNS (aVNS).

## Current clinical uses of VNS

### Methods of stimulating VN

**Figure 1:**
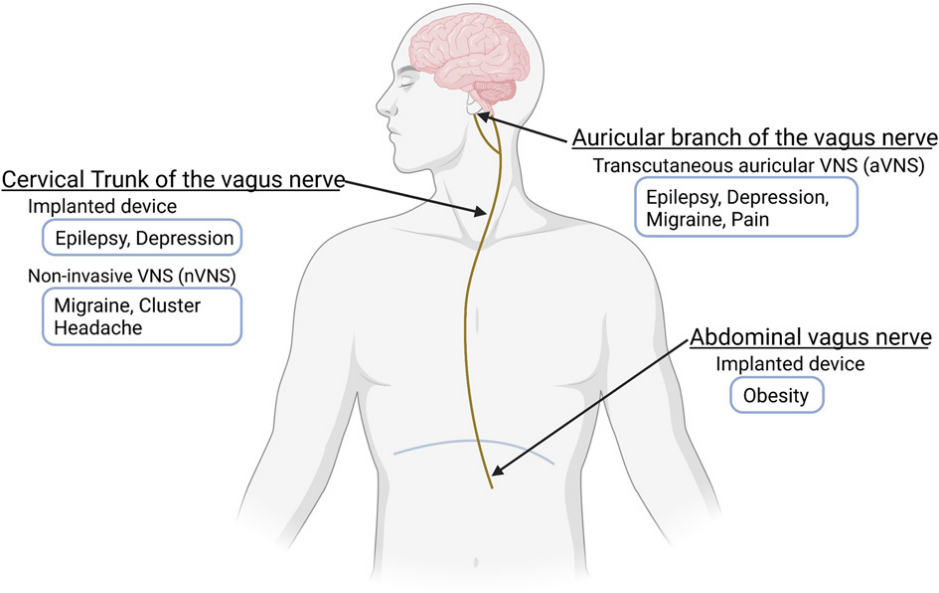
FDA-approved clinical uses of VNS The FDA approved an implanted cervical VNS device consisting of the pulse generator, lead wire, and external remote controls for epilepsy and depression, and non-invasive VNS device (applied to the neck) for migraine and cluster headache. In transcutaneous aVNS, the auricular branch of VN is stimulated with electrodes placed on the skin of the external ear in a non-invasive manner for epilepsy, depression, migraine, and pain. The FDA also approved the Maestro Rechargeable System, with implanted electrode wires at the subdiaphragmatic level, for the treatment of obesity.

VNS is a general term that describes any technique that stimulates the VN (Figure 1). Different indications require different approaches to target the VN, as will be discussed subsequently. Most commonly, VNS involves stimulating the left cervical VN by surgically implanting a pulse generator device. The left as opposed to the right cervical VN is targeted to minimize cardiac effects including bradycardia. Right cervical VNS has been used mainly in the context of heart failure [10,11]. In early trials, a programmable device was implanted into the right chest and connected to the right cervical VN. This device was designed to affect cardiac function by preferentially activating vagal efferent fibers. The subdiaphragmatic VN can also be targeted by implantation of electrodes on the ventral and/or dorsal vagal trunks below the diaphragm. This approach has been investigated for its effects on food intake and in the treatment of obesity [12–16]. Transcutaneous VNS is a non-invasive alternative to the invasive implantable VNS devices. Most commonly, surface electrodes are placed on the external ear to target the auricular branch of the VN (described in depth in later sections). Another form of transcutaneous VNS targets the cervical VN in the neck and has been investigated in various disorders including headaches [17–19].

### Epilepsy

In 1997, the first VNS device was approved by the FDA for patients, aged 12 and older, with medically refractory partial onset seizures. After preclinical studies demonstrating encouraging results in dogs [20] and monkeys [21], two pilot, single-blind trials of an implantable VNS device were initiated [22]. Following this, two multicenter, prospectively randomized, double-blind trials began [23,24]. Sixty-seven patients were randomized to receive high or low VNS treatment for 14 weeks. At the end of the study period, 38.7% of patients receiving high VNS achieved at least 50% reduction in seizure frequency compared with 19.4% of patients receiving low VNS, and the device was well-tolerated [23]. These trials prompted the initial approval of VNS by the FDA and since this time, the approval has been expanded to include children as young as 4 and to include additional stimulation devices. Additionally, while initially used for focal seizures, it is now also being used to treat generalized seizures [25].

Implantable VNS devices are now widely used in the treatment of drug-resistant epilepsy for patients who are not eligible for epilepsy surgery. The device consists of implantable elements including the pulse generator and lead wire and external remote controls that allow the patient to stop the stimulation or trigger a pulse [26]. These devices generally last 6–10 years depending upon the amount of use and settings. Although efficacious, VNS is not first-line therapy, and side effects, including cough, dysphonia, surgical site infection and hoarseness, are not uncommon. Other surgical complications have been reported including postoperative hematoma, infection, and vocal cord palsy, in addition to hardware-related complications such as lead fracture [27].

A review encompassing 30 years of data suggests that VNS achieves optimal efficacy at 6 months of treatment with a 50–100% reduction in seizure frequency [26]. A large retrospective study using patient outcome registry data from over 4000 patients examined the effects of VNS for the treatment of epilepsy based on patient age, epilepsy duration, and seizure type [28]. After 3 months, a 46% reduction in seizure frequency was achieved with 44% of patients having at least a 50% reduction. These positive outcomes were increased at the 1- and 2-year follow-ups. Patients less than 18 years old and those with a history of epilepsy of less than 10 years achieved a more favorable response to VNS therapy compared with older adults and those with a longer disease history. Additionally, the largest benefit was achieved in those with predominantly simple-partial seizures [28].

Despite many years of its use and numerous preclinical and clinical studies demonstrating its clear therapeutic effect, the exact mechanism by which VNS controls epilepsy has not been elucidated [29]. Multiple mechanisms have been proposed and have mostly focused on the ability of VNS to reduce excitability in various brain regions, its neuroelectrophysical role, and its effects on the release of neurotransmitters [30,31]. For example, studies have suggested that the mechanism of benefit is due to the increased norepinephrine release from the locus coeruleus [32,33]. More recent evidence has demonstrated the role of inflammation in epilepsy. This has led to the speculation that the protection by VNS in epilepsy is in part mediated by its anti-inflammatory effect [31]. Despite the proven benefit of VNS in epilepsy, more work is needed to understand its mechanism of protection. Identifying the precise mechanism of action of VNS in epilepsy may help identify responders vs. non-responders and tailor the treatment to those who will be most likely to benefit. Interestingly, a recent study used a statistical model based on preoperative heart rate variability (HRV) to predict which patients would be suitable for VNS [34]. Vagal efferents innervate the sinoatrial node and the atrioventricular node to control heart rate [35]. HRV is an indirect reflection of the heart’s autonomic function and VNS has been shown to induce acute increases in HRV [36]. The investigators found that perioperative HRV indices, recorded during sleep, could better predict response to VNS therapy, compared with measurements recorded during the awake state [34].

### Depression

Patients with epilepsy treated with VNS were noted to have improvement in mood [37]. In 2000, Rush et al. conducted the first study investigating the effects of VNS on patients with depression and without epilepsy [38]. In 2005, the FDA approved a VNS device, made by Cyberonics, for treatment-resistant depression based on the results from a 12-week randomized controlled acute phase trial, a 12-month prospective naturalistic study, and a prospective 1-year comparison of VNS with treatment as usual for treatment-resistant depression [39–41]. Since this time, more studies have been conducted supporting the efficacy of VNS in depression. A study by Bajbouj et al. demonstrated that 53.1% of patients with chronic treatment refractory depression met the response criteria of a 50% reduction in the Hamilton Rating Scale for Depression (HRSD28) score after treatment with VNS [42]. A recent meta-analysis found that adjunctive VNS in treatment-resistant depression appears to be effective, relatively safe, and well-tolerated [43]. However, in order to gain more widespread acceptance among clinicians, larger trials are needed. A large 5-year randomized controlled trial is currently underway and the results obtained from this will likely help in clarifying the role of VNS in depression [44].

Multiple techniques including functional magnetic resonance imaging (fMRI), positron emission tomography (PET), and single photon emission computed tomography (SPECT) have been employed to elucidate the mechanism of action of VNS in depression [45]. These mechanisms can be divided into acute and chronic effects. Conway et al., using oxygen 15-labeled water PET, demonstrated that acute VNS produced changes in mean cerebral blood flow in multiple regions implicated in depression [46]. Increases in blood flow were seen in the bilateral orbitofrontal cortex, bilateral anterior cingulate cortex, and right superior and medial frontal cortex while decreases were seen in the bilateral temporal cortex and right parietal area. Additionally, Nahas et al. used blood oxygenation level-dependent (BOLD) fMRI to study the sustained effects of VNS [47]. In the acute setting, VNS led to activation in both the right medial prefrontal cortex and the right anterior insular cortex. However, at approximately 30 weeks, this activation switched to deactivation which correlated with a decrease in depressive symptoms [45,47].

The effect of VNS on noradrenergic and serotonergic neurons has also been proposed to be part of its beneficial effect. In multiple studies, VNS has been shown to increase noradrenaline concentrations and improve noradrenaline and serotonergic neurotransmission in brain regions important in depression [48–50]. Furthermore, Grimonprez et al. evaluated the anti-depressant effects of VNS after lesioning of noradrenergic neurons from the locus coeruleus in rats during the forced swim test (to assess depression-like behavior) or open field test (to assess locomotor activity) [51]. While VNS reduced the immobility time during the forced swim test, this effect was not seen with lesioning of the locus coeruleus, suggesting that noradrenergic neurons from the locus coeruleus contribute to the anti-depressant effect of VNS. While anti-depressants can increase neurotransmitter release and affect the sensitivity of inhibitory receptors, VNS appears to alter the baseline firing rate of noradrenergic and serotonergic neurons and thus has a distinct mechanism. Additionally, an effect of VNS on the dopaminergic system has been suggested. For example, VNS treatment of patients with treatment-resistant depression increased cerebrospinal fluid levels of homovanillic acid, a dopamine metabolite [52]. Clearly much work has been done to decipher the anti-depressant effects of VNS, however, there are still many unknowns and more studies are needed to gain a more precise understanding.

### Obesity

Inspired by the finding that patients being treated with VNS for epilepsy experienced weight loss, VNS was investigated for its effects on body weight and food intake and in the treatment of obesity [12,13,53,54]. Furthermore, in a study of patients with depression treated with cervical VNS for 2 years, participants were observed to have a decrease in weight unassociated with mood changes [55]. Additionally, altered vagal activity had been demonstrated in rodent models of obesity [56,57].

A study investigating the mechanism of VNS in treating obesity in rats demonstrated that VNS delayed gastric emptying by releasing anorexigenic hormones and enhancing vagal activity [58]. These hormones included glucagon-like peptide-1, polypeptide YY, and pancreatic polypeptide. In this study, electrode wires were implanted in the subdiaphragmatic vagal nerves on both the left and right and externally at the back of the neck. The mechanism for the increase in these hormones with VNS was not determined. In another preclinical study, PET was employed to determine the effect of VNS on eating behavior in pigs [59]. After 5 weeks of VNS, food intake decreased and PET demonstrated activation of central dopaminergic reward areas with VNS. Because gastric compliance and emptying were unaltered, the authors suggest that this effect was not due to vagus efferents [59,60].

These data suggested that modulating the VN could represent a possible therapy for obesity. Most of these preclinical and clinical studies used low frequency (<30 Hz) VNS. While low-frequency VNS elicits action potentials in vagal fibers, high frequency reversibly blocks action potentials [61,62]. The use of high-frequency (kilohertz) stimulations that reversibly block conduction to the subdiaphragmatic VN has been investigated in the treatment of obesity. In 2011, the ReCharge trial, a multicenter randomized double-blind clinical trial evaluating the effectiveness of the Maestro Rechargeable System, began (NCT01327976). This device delivers intermittent, electrical blocking signals to the anterior and posterior trunks of the intra-abdominal VN. A total of 162 and 77 obese individuals were randomized to the vagal nerve block (5 kHz) or sham device group, respectively. After 12 months, participants in the vagal nerve block group had a statistically significant greater weight loss (24.4%) compared with those in the sham group (15.9%) [15]. However, this did not reach the primary efficacy endpoint of at least a 10% greater weight loss reduction. Still, based on these results and the sustained results after 18 months, in 2015, the FDA approved the use of this device in the treatment of obesity [16]. It is important to note that, while these data suggest efficacy, vagal blockade does not play a main role in the treatment of obesity and more trials and long-term follow-up studies will be needed before devices such as the Maestro Rechargeable System are incorporated into clinical practice.

Additionally, the effect of transcutaneous auricular VNS (TENS) in the treatment of obesity is currently under exploration in human trials (NCT05230628, NCT04926415). In a randomized triple-blind trial, 150 obese participants will receive either active TENS, at 25 Hz, three to four times a day for 10 min, 30 min before the main meals, or sham stimulation, for 6 months (NCT05230628). Primary outcome measures include changes in percentage of body fat, BMI, and waist circumference.

### Headache

Several case reports demonstrated improvement in migraine in patients with refractory epilepsy treated with VNS. Additionally, it had been shown that VNS could modulate pain. Together, these findings suggested the possible utility of VNS in the treatment of migraine. However, the invasive nature of surgically implanted devices limited VNS’s widespread adoption despite demonstrated benefit. GammaCore is a patient-controlled handheld non-invasive transcutaneous device designed to deliver electrical stimulation to the cervical VN during acute migraine attacks. In 2012, a pilot study was initiated which demonstrated the potential tolerability and efficacy of the non-invasive VNS (nVNS) device in the treatment of migraine [17]. Following this, in a multicenter study, patients with high-frequency episodic migraine or chronic migraine were treated with nVNS during acute migraine attacks [18]. Over the course of 2 weeks, patients self-delivered two 120-s doses of electrical stimulation, 3 min apart, at the onset of a migraine attack. In this population, 56.3% reported pain relief at 1 h post-stimulation and 64.6% at 2 h post-stimulation. In the PREVA trial, patients with chronic cluster headache were randomly assigned to receive adjunctive prophylactic nVNS or standard of care (SOC) alone [19]. In this trial, patients prophylactically administered stimulations 10 h apart with the option of administering additional stimulations during acute headache attacks. Patients treated with nVNS and SOC, over the course of 4 weeks, reported a greater reduction in the number of attacks during the study period compared with those treated with SOC alone. In 2018, nVNS was approved by the FDA for migraines and then in 2019 for cluster headaches. More recently, the FDA approved the use of an nVNS device for children aged 12–17 with migraine. The mechanism of VNS’s beneficial effect in headaches is not well understood. In a review, Silberstein et al. suggest that VNS has effects on four main areas: the autonomic system, neurotransmitters, cortical spreading depression, and nociception [63]. Recently, Hu et al. investigated whether the anti-nociceptive effect of VNS involved opioidergic mechanisms. In this preclinical model, the authors provide evidence that VNS engages the δ opioid receptor (DOR) [64]. Together, these data suggest that VNS may provide an efficacious non-pharmacological approach to treating migraines and cluster headaches.

## Potential uses and mechanisms of VNS

Much excitement has focused on using VNS as an anti-inflammatory therapy in treating diseases including diabetes, Alzheimer’s disease, cardiovascular disease, and arthritis. VNS can attenuate the inflammatory response by activating a neuroimmune circuit known as the cholinergic anti-inflammatory pathway (CAP). The CAP, first described by Kevin Tracey, is the efferent limb of the inflammatory reflex [65,66]. Two immune cell types, β_2_ adrenergic receptor positive CD4+ T cells and α7 nicotinic acetylcholine receptor (α7nAChR) expressing macrophages, play an important role in the CAP. Firing of the efferent VN initiates the CAP. This signal is then transmitted to the splenic nerve which releases norepinephrine which binds to β_2_ adrenergic receptors on choline acetyltransferase-positive T cells in the spleen. Acetylcholine is released from these cells and binds to α7nAChRs on macrophages. This ultimately results in the suppression of inflammation by reducing cytokine production in the spleen. The initial finding that activation of cholinergic neurons can decrease inflammation instigated the exploration of the use of VNS in inflammation-mediated diseases. Recent evidence suggests that VNS can also activate other neuroimmune circuits to decrease inflammation, which has been reviewed elsewhere [67,68]. Preliminary evidence suggests that VNS may be applied as an anti-inflammatory treatment for a broad range of diseases, a few of which will be reviewed here.

### Inflammatory bowel disease

The VN connects the CNS to the digestive system through the brain–gut axis. Vagal afferents contain chemoreceptors, mechanoreceptors, thermoreceptors, and osmoreceptors. They can detect the status of the gastrointestinal tract and communicate this information to the CNS. Vagal efferent fibers, originating in the brain, synapse with second order post-ganglionic neurons located in the digestive wall [69].

Inflammatory bowel disease (IBD) is a group of inflammatory conditions that involve the colon and small intestine. It is generally divided into ulcerative colitis (UC) and Crohn’s disease (CD). Currently, there is no cure for IBD. Treatment often involves pharmacologically targeting pro-inflammatory cytokines or surgery. Most commonly, IBD is treated with anti-TNF agents, however, these face numerous issues including side effects, loss of response, low patient compliance, and high cost [69]. Thus, additional anti-inflammatory therapies are of great interest [69].

In 2003, Miceli and Jacobson, showed that administration of anticholinesterase drugs improved colitis in a CD model [70]. It was later shown, in a mouse model, that vagotomy exacerbated colitis [71]. Low vagal tone was shown to be associated with high plasma TNF-α levels [72]. In a cohort study, vagotomy was associated with an increased risk of developing IBD, enforcing a beneficial role of vagal tone in IBD [73]. Kevin Tracey’s group demonstrated that VNS during endotoxemia decreased TNF-α production by splenic macrophages [74]. Additionally, dysbiosis is a common feature in IBD and, through the CAP, the VN could affect the intestinal microbiota [75].

These data suggested an anti-inflammatory role of the VN during digestive inflammation and laid down the groundwork for the potential use of VNS in TNF-mediated chronic inflammatory diseases. In multiple studies, it was shown that chronic VNS improved colitis in rats [76–78]. The first clinical trial involving treatment of patients with IBD with VNS began in 2012 [69,79,80]. Nine patients were recruited to receive VNS continuously for 12 months. Clinical, biological, and autonomic markers were measured over the course of the study period. Five of the seven patients who completed the study achieved remission and five were shown to have a decrease in the CD endoscopic score of severity. Additionally, VNS was shown to restore vagal tone in these patients. These findings were supported by a study conducted by d’Haens et al. (NCT02311660) who found that VNS monotherapy or as adjunctive therapy improved clinical and endoscopic markers in half of IBD patients [81]. For 2 weeks, daily VNS for 60 s was initiated. From 4 to 6 weeks, stimulation was increased to 5 min daily along with an increase in output current. If the Crohn’s Disease Activity Score (CDAI) did not improve by week 8, stimulations increased to four times daily for the remainder of the 16-week study period. Response to therapy was assessed based on CDAI, fecal calprotectin levels, and Simple Endoscopic Score for CD. While these data are promising, more trials are needed to assess the clinical efficacy of VNS in IBD. Recently, in 2021, a pilot study was initiated investigating the effects of transcutaneous VNS in CD. In this open-label, single-arm trial, patients will self administer VNS to the cervical VN three times per day for 16 weeks, using a handheld device (NCT05165108). Changes in CDAI, fecal calprotectin levels, cytokines, and HRV will be assessed at the end of the study period.

### Kidney disease

In 2010, Hoeger et al. investigated the effects of VNS on brain death-induced inflammation. Specifically, the authors performed VNS on brain-dead kidney transplant donor rats and assessed its impact on graft outcome [82]. VNS resulted in improved renal function in the transplant recipient. This was followed by a study demonstrating that VNS on brain-dead donor rats also decreased chronic allograft nephropathy in recipients [83]. Thus, these studies suggested a role for VNS in kidney pathology.

In 2016, another study was conducted to gain a more mechanistic insight into the effects of VNS on acute kidney injury (AKI) [84]. Prior preclinical studies had demonstrated that pulsed ultrasound administered before renal ischemia–reperfusion injury (IRI) could attenuate injury [85] leading to the hypothesis that VNS could protect against renal IRI similar to ultrasound treatment. Indeed, Inoue et al. demonstrated that VNS ameliorates kidney IRI and showed that this effect was dependent upon the CAP [84]. Of note, VNS was effective when delivered 24 h but not 10 min before IRI. From a clinical perspective, this study suggested that VNS could be employed prophylactically in situations in which the patient is at high risk of developing AKI. This was followed by a study demonstrating that VNS administered 24 h after cisplatin treatment was protective against kidney injury which was dependent upon the CAP [86]. Preclinical studies have clearly demonstrated a protective role of VNS in kidney injury and clinical trials are now warranted to determine its therapeutic benefit in patients.

Recently, Hilderman and Bruchfeld conducted a pilot clinical trial involving VNS treatment in hemodialysis patients [87]. The authors hypothesized that VNS would suppress inflammation and alter HRV in hemodialysis patients. Twelve hemodialysis patients were treated with a minimally invasive oscillating device before dialysis three times a week for 4 weeks. In the present study population, VNS did not significantly change cytokine levels, nor did it alter HRV. However, there were many limitations to the present study. Firstly, VNS was not administered daily due to practical limitations. Additionally, the minimally invasive device used may not have been optimal in this context. Finally, the small sample size and lack of control group hinders interpretation of the results. Clearly more research will be needed to assess the efficacy of VNS in hemodialysis patients and in other kidney pathologies.

### Rheumatoid arthritis

Rheumatoid arthritis (RA) is a chronic autoimmune disease characterized by synovial inflammation and progressive erosions of bone and cartilage. Inflammation plays a critical role in the pathogenesis. Pro-inflammatory cytokines are released and thus pharmaceutical agents that suppress this response, including glucocorticoids, methotrexate, and monoclonal antibodies, have been the focus of treatment [88]. Specifically, anti-TNF drugs are widely used. Despite advances in therapeutic agents, there is no mainstay of treatment and many patients do not respond adequately.

Koopman et al. demonstrated that stimulating the inflammatory reflex with an implantable VNS device can modulate TNF and other cytokines [88]. As part of the study, 18 patients with RA were enrolled to receive VNS for 60 s up to four times daily. Peripheral blood was collected on day 42 which showed a decrease in TNF levels with VNS. Stimulation was stopped for 14 days during which time TNF levels increased. Restarting VNS on day 56 led to another decrease in TNF by day 84. This suggested that active stimulation is needed to decrease TNF levels and withdrawal of treatment may worsen disease. RA clinical disease severity also significantly improved with active VNS. The present study was followed by a pilot study evaluating the safety and efficacy of a novel miniaturized VNS device in patients with multidrug refractory RA [89]. This was a two-stage study; stage 1 was open label and three participants received active stimulation for 1 min daily. In stage 2, 11 patients were randomly assigned to receive active stimulation (1 min) once daily, active stimulation (1 min) four times daily, or sham stimulation for 12 weeks. Investigators and participants were blinded during stage 2. The device was safe and well-tolerated and, similar to earlier findings, reduced biomarkers and clinical signs of RA. Furthermore, in a recent open-label single-center pilot study, transcutaneous non-invasive cervical VNS was delivered to 36 patients with RA of either high- or low-disease activity [90]. Outcome measures included changes in Disease Activity Score based on 28-joint count–C-reactive protein (DAS28-CRP), cardiac vagal tone, and pro-inflammatory cytokines. In the 16 patients with high disease activity, VNS reduced the levels of DAS28-CRP, CRP, and interferon-γ after 4 days of treatment. These data suggest a role for VNS as a safe and efficacious anti-inflammatory treatment for RA and support the undertaking of larger clinical trials.

The RESET-RA study is an ongoing multicenter, randomized, sham-controlled blinded trial in which 250 participants will be recruited to receive either active VNS or sham stimulation (NCT04539964). Participants with moderate-to-severe RA who have failed to respond to biologic agents or DMARDs are eligible. All participants will receive an implantable VNS device on the left cervical VN. Stimulations will be delivered for 1 min daily for 12 weeks. At week 12, after endpoint assessments, participants in the control group will receive active stimulations. All patients will be monitored in an open label 180-week follow-up. The primary endpoint is an American College of Rheumatology (ACR) 20 response at 12 weeks.

### Stroke

Motor function is often significantly impaired following ischemic stroke, a leading cause of disability worldwide [91]. Although multiple motor rehabilitation methods, which can promote major neural plasticity, have been developed, significant deficits often persist following stroke. Pharmacological agents acting on neurotransmitters have been shown to generate neural plasticity and potentiate motor rehabilitation treatments [92]. Similarly, VNS can modulate the release of neurotransmitters and enhance neural plasticity. For example, VNS, through muscarinic receptors, influences cortical synchrony and excitability [93]. Additionally, repeatedly pairing VNS with a specific movement reorganizes the primary motor cortex and increases the cortical representation of that movement [94].

In 2014, VNS was delivered during motor rehabilitation in a rat model of stroke to test the hypothesis that this could improve recovery of motor function [95]. Indeed, the authors found that VNS repeatedly paired with successful upper forelimb movements improved recovery after stroke compared with rehab alone. VNS delivered after rehab did not have an effect compared with rehab alone. The present study provided initial indication that VNS could represent a novel method to improve stroke rehabilitation and has since been followed-up with trials in humans. In 2016, Dawson et al., in a pilot clinical study, demonstrated the safety and feasibility of VNS paired with upper-limb rehabilitation after ischemic stroke [96]. Participants were implanted with a VNS device on the left cervical VN. While the patients performed a task involving the upper limb, a therapist manually delivered a stimulation using a push button on a wireless external device. In the recent VNS-REHAB trial, 108 patients with moderate­-to-­severe loss of arm function after ischemic stroke were enrolled and were randomly assigned to receive active VNS or sham stimulation paired with intensive motor rehabilitation for 6 weeks [97]. The primary outcome was the change in Fugl­Meyer Assessment Upper Extremity (FMA­UE) score. After 6 weeks, the mean FMA­UE increased by 5.0 points (SD 4.4) in the VNS group and 2.4 points (3.8) in the control group. After 90 days, a clinically meaningful response was seen in 47% of patients in the VNS group compared with 24% of patients in the sham group. Interestingly, the improvement with VNS was observed even in patients who suffered from stroke many years prior (mean time since stroke was 3.1 years). This well executed trial provides strong support for the efficacy of VNS in patients with stroke, however, many questions still remain. Neuroplasticity may vary by individual, particularly by age and sex, and thus larger studies with subgroup analyses should be conducted to investigate the effect this may have. Additionally, longer follow-up studies will be needed to assess whether long-term synaptic changes occur with stimulation. The excitement for the potential of VNS to improve motor recovery following stroke is growing and is now supported by both preclinical and clinical data.

### Heart failure

NECTAR-HF was a randomized sham-controlled trial designed to evaluate whether right cervical VNS would attenuate cardiac remodeling, improve cardiac function, and increase exercise capacity in symptomatic heart failure patients with severe left ventricular systolic dysfunction [98,99]. All patients (*n*=96) were implanted with a VNS system and randomized in a 2:1 ratio to receive therapy (VNS) or control (sham stimulation) for a 6-month period. The primary endpoint was the change in left ventricular-end systolic diameter. Secondary endpoints included other echocardiography measurements, exercise capacity, quality-of-life assessments, 24-h Holter-derived indices of autonomic nerve modulation, and circulating biomarkers. There were statistically significant improvements in quality of life, New York Heart Association (NYHA) class, and the SF-36 Physical Component in the VNS group although the other endpoints were not different between the groups. INOVATE-HF was a multinational, randomized trial involving 85 centers including patients with chronic heart failure, NYHA functional class III symptoms, and ejection fraction ≤ 40% [100]. Patients (*n*=707) were assigned to device implantation to provide VNS or continued medical therapy in a 3:2 ratio and were followed-up for a mean of 16 months. A nerve stimulation cuff was implanted on the right cervical VN in addition to a transvenous lead in the right ventricle to detect ventricular activation. There was no difference in the primary efficacy outcome (composite of death from any cause or first event for worsening heart failure). Quality of life, NYHA functional class, and 6-min walking distance were better in the VNS group, but left ventricular end-systolic volume index was not different. Thus, these clinical trials failed to show beneficial effects of VNS on death, heart failure events, or cardiac remodeling/function in chronic heart failure patients although quality-of-life measures were significantly improved by VNS.

## Advances in VNS

### aVNS

While cervical VNS has demonstrated to be a feasible and efficacious treatment in various diseases, it is invasive and thus poses a risk to individuals. Therefore, the development of a non-invasive approach with similar efficacy has been of great interest.

The external ear is the only location where the vagus sends its peripheral branch, the auricular nerve (aVN). The antihelix, cavity of concha, tragus, crus of helix, and crura of antihelix of the ear are partly innervated by the aVN, while the cymba concha is innervated exclusively by the aVN [101]. As with the VN, the aVN is composed of myelinated A and B fibers as well as unmyelinated C fibers [102]. The ear also contains endings of other nerves including the great auricular nerve, the auriculotemporal nerve, and the lesser occipital nerve.

Although auricular acupuncture has been used in eastern medicine going back 2500 years [103], the potential to electrically stimulate the auricular nerve was first demonstrated in healthy subjects in 2003 [104]. Stimulation of the aVN sends signals directly to the brainstem and thus, the aVN provides an external gateway to brain. Both transcutaneous and percutaneous aVNS techniques exist and are approved for treatment of select diseases [102]. In transcutaneous aVNS, the afferent VN endings are stimulated with electrodes placed on the skin of the external ear. Strong currents are needed to pass through the skin and a relatively large surface area is stimulated. Percutaneous aVNS, on the other hand, is a minimally invasive technique in which electrodes penetrate the skin of the ear in the regions of the aVN. The needles can be focused to the target region, circumventing the large surface area required in transcutaneous aVNS.

The NSS-2 BRIDGE Device is a percutaneous neurostimulator that recently received FDA clearance for the treatment of symptoms resulting from opioid withdrawal. The device, which is placed behind the patient’s ear, stimulates cranial nerves V, VII, IX and X and the occipital nerves. A recent study assessed the role of the NSS-2 BRIDGE Device in managing Post-Operative Pain in Total Knee and Hip Arthroplasties, Bariatric, and Kidney Transplant Surgeries (NCT03834142). For each surgery, ten patients were recruited to receive the device and were compared with ten historical controls who had received SOC. The primary endpoint was a reduction in opioid requirement 24 h after surgery. The NSS-2 BRIDGE Device reduced the oral morphine equivalent (OME) by 75.4% and reduced pain by 41.5% 24 h after surgery in kidney donor patients [105]. In bariatric patients, the NSS-2 BRIDGE Device reduced the OME by 60.2% and pain by 28% at 24 h following laparoscopic Roux-en-Y gastric bypass surgery [106]. A recent study evaluated the use of a transcutaneous aVNS device in pediatric patients with relapsing nephrotic syndrome (FRNS) and steroid-resistant nephrotic syndrome (SRNS) [107]. The device used was the transcutaneous electrical nerve stimulation (TENS) unit that was attached to the patients’ ears via an ear clip to left cymba concha. Guardians performed transcutaneous aVNS on their child 5 min daily for 26 weeks. All patients with FRNS remained relapse free during the study period. In three of the four patients with SRNS, TENS reduced the urine protein:creatinine below the nephrotic range. Additionally, there was a significant decrease in serum TNF levels compared with baseline. The results of the present study warrant larger trials studying the efficacy of transcutaneous aVNS in the treatment of nephrotic syndrome.

The ability of aVNS to modulate multiple central brain structures has been evaluated using various techniques including fMRI, extracellular recordings, EEGs, and transcranial magnetic stimulation [101]. In general, the neurophysiological effects of aVNS are considered to be similar to that of VNS [101]. The aVN directly projects to the NTS and thus affects both the central and autonomic nervous systems, resulting in widespread and systemic effects [108]. As discussed above, VNS has an anti-inflammatory effect. Similarly, aVNS reduced pro-inflammatory cytokine levels in humans [109] and increased norepinephrine levels in rats [110]. Additionally, aVNS may exert its actions through the CAP, as shown in endotoxemic rats [111]. Thus, like VNS, aVNS shows promise in the treatment of inflammatory conditions. The anti-nociceptive effects of cervical VNS have also been similarly demonstrated in both preclinical and clinical studies of aVNS (as reviewed [101]). In addition, transcutaneous electrical auricular stimulation was reported to reduce cardiac remodeling after myocardial infarction in dogs, an effect similar to that of cervical VNS [112]. Although the authors did not investigate the mechanism behind this effect, they state that the stimulation produced the same effects as cervical VNS on vagal efferent fibers. For a deeper review of the evidence for the effects of aVNS, the reader is referred to the comprehensive review by Kaniusas et al. [101].

Currently, aVNS is being explored in many of the diseases that VNS has been used to treat. Importantly, many of these studies and clinical trials have shown similar efficacy of aVNS compared with invasive VNS. For example, aVNS and VNS both decreased epileptic seizure activity in rat models [113]. The anti-seizure effect of aVNS was shown to have a similar duration of effect compared with that of invasive cervical VNS. There are also numerous ongoing clinical trials investigating the use of aVNS in a broad range of applications. For example, it is being examined in the treatment of fibromyalgia (NCT04260906), alcohol withdrawal (NCT04159909), and for kidney transplant recipients (NCT04256837). It is even being used paired with bottle feeding to improve feeding in newborn infants [114–116] and in a trial to determine whether it can influence consciousness (NCT04065386).

Although aVNS, compared with cervical VNS, offers the clear advantage of being less invasive, it still has its own shortcomings. For example, the stimulation may cover a large area of the auricle, particularly with transcutaneous aVNS, and thus additional auricular nerves may be stimulated. This is complicated by the controversy surrounding the precise innervation of the auricle, as only few studies have been conducted in this area. The concha is generally used as the ear target for aVNS; however, some suggest that stimulation of the tragus may be more advantageous [117]. Stimulation of different regions of the ear can produce different effects which may impact a study’s results and reproducibility [108,118]. Additionally, multiple reflexes can be triggered during aVNS, particularly the Arnold’s ear cough reflex, but also the ear-gag, ear-syncope, and ear-lacrimation reflexes [101]. Finally, aVNS can cause many of the same side effects of VNS such as dizziness, headache, and stimulation site skin irritation [119].

The enthusiasm for the potential applications of aVNS are clear. Whether this therapy can live up to these high expectations across a multitude of disciplines remains to be determined.

### Optogenetics

VNS simultaneously excites all neurons surrounding the electrode tip and thus it is nonspecific and lacks spatial resolution. Optogenetics is a neuromodulatory technique that uses light to manipulate cells, typically neurons, which have been genetically manipulated to express light-sensitive opsins [120]. Optogenetics is a powerful tool and the use of optogenetics in combination with VNS has been under investigation in a few select diseases thus far. For example, with the use of optogenetics, we recently performed selective VNS (efferent vs. afferent fibers) and found that stimulation of either vagal efferent or afferent fibers was sufficient to protect mice from renal IRI [121]. Additionally, it has been unclear whether the benefit of VNS in HF in clinical trials is due to the recruitment of efferent or afferent fibers or both. In a few preclinical studies, selective stimulation of specific vagal fibers by optogenetics demonstrated that vagal efferent fiber stimulation may be crucial in the beneficial effect of VNS in HF and can reduce afferent fiber-related side effects [122–124]. The authors suggest that it will be necessary to develop a VNS device with selective stimulation of a subset of vagal fibers if VNS is to be applied in the treatment of HF.

Although therapeutic application of optogenetics is not yet a reality, preclinical studies utilizing optogenetics have the potential to identify a specific neural circuit important in mediating the protective effect of VNS and greatly enhance our understanding of the mechanisms of VNS.

## Conclusion

VNS is an effective therapy that has already received FDA approval in the treatment of epilepsy, depression, migraines and cluster headaches, and in the abdomen for obesity. Preclinical studies and clinical trials have suggested a potential benefit in an even broader range of diseases. FDA approval for the use of VNS in the treatment of some of these diseases may be on the horizon. Before then, however, larger clinical trials and continued investigations into the mechanism of VNS’s benefit in each disease will be needed. The non-invasive nature of aVNS may help to expand and accelerate the application of VNS in the treatment of more diseases, although this will require further studies. Finally, optogenetics shows great promise in aiding mechanistic investigations of VNS.

## Data Availability

Data are not present in this review article.

## Open Access

Open access for this article was enabled by the participation of University of Tokyo in an all-inclusive *Read & Publish* agreement with Portland Press and the Biochemical Society.

## Abbreviations

AKI: acute kidney injury
aVNS: auricular vagus nerve stimulation
BMI: body mass index
CAP: cholinergic anti-inflammatory pathway
CDAI: Crohn’s disease activity score
CD: Crohn’s disease
CNS: central nervous system
DAS28-CRP: disease activity score based on 28-joint count–C-reactive protein
DMARD: disease-modifying antirheumatic drug
DMN: dorsal motor nucleus
EEG: electroencephalogram
FDA: Food and Drug Administration
HF: heart failure
FMAUE: Fugl­-Meyer assessment upper extremity
fMRI: functional magnetic resonance imaging
FRNS: frequently relapsing nephrotic syndrome
HRV: heart rate variability
IBD: inflammatory bowel disease
IRI: ischemia–reperfusion injury
nVNS: non-invasive VNS
NYHA: New York Heart Association
OME: oral morphine equivalent
PET: positron emission tomography
RA: rheumatoid arthritis
SOC: standard of care
SRNS: steroid-resistant nephrotic syndrome
TENS: transcutaneous electrical nerve stimulation
TNF: tumor necrosis factor
UC: ulcerative colitis
VNS: vagus nerve stimulation
α7nAChR: α7 nicotinic acetylcholine receptor
